# Human papillomavirus vaccine initiation among 9–13-year-olds in the United States

**DOI:** 10.1016/j.pmedr.2015.10.003

**Published:** 2015-10-21

**Authors:** Kelly L. Donahue, Kristin S. Hendrix, Lynne A. Sturm, Gregory D. Zimet

**Affiliations:** Indiana University School of Medicine, Dept. of Pediatrics, 410 West 10th Street, Suite 1001, Indianapolis, IN 46202, USA

**Keywords:** ACIP, Advisory Committee on Immunization Practices, Early adolescence, Healthcare providers, Immunization

## Abstract

The quadrivalent and 9-valent human papillomavirus (HPV) vaccines are licensed for administration among 9–26-year-old males and females, with routine vaccination recommended for 11–12-year-olds. Despite the availability of the vaccine at younger ages, few studies have explored vaccine uptake prior to age 13, and national HPV vaccination surveillance data is limited to 13–17-year-olds. Our objective was to examine rates and predictors of HPV vaccine initiation among 9–13-year-olds in the United States. A national sample of mothers of 9–13-year-olds in the United States (*N* = 2446) completed a 2014 Web-based survey assessing socio-demographic characteristics, child's HPV vaccination history, provider communication regarding the vaccine, and other attitudes and behaviors pertaining to vaccination and healthcare utilization. The main outcome measure was child's initiation of the HPV vaccine (i.e., receipt of one or more doses). Approximately 35% of the full sample and 27.5% of the 9–10-year-olds had initiated HPV vaccination. Females were more likely than males to have initiated HPV vaccination by the age of 13 but not by younger ages. Strength of health provider recommendation regarding HPV vaccination was a particularly salient predictor of vaccine initiation. Approximately a third of children may be initiating the HPV vaccine series before or during the targeted age range for routine administration of the vaccine. Because coverage remains below national targets, further research aimed at increasing vaccination during early adolescence is needed. Improving providers' communication with parents about the HPV vaccine may be one potential mechanism for increasing vaccine coverage.

## Highlights

• Approximately 35% of 9–13-year-olds had initiated the HPV vaccine series.
• A quarter of 9–10-year-olds had initiated the HPV vaccine series.
• At age 13, females were more likely than males to have initiated HPV vaccination.
• No gender difference in HPV vaccine initiation was found prior to age 13.
• Provider recommendation was a particularly salient predictor of HPV vaccination

## Introduction

Human papillomavirus (HPV) is a highly prevalent sexually transmitted infection affecting both males and females (Hariri et al., 2011, Forhan et al., 2009, Giuliano et al., 2008). HPV is the primary cause of cervical cancer and leading cause of other anogenital and oropharyngeal cancers, in addition to causing genital warts (Schiffman et al., 2007, Munoz et al., 2004, Watson et al., 2008, Jayaprakash et al., 2011). Vaccination provides effective protection against HPV and its associated adverse health outcomes (Baandrup et al., 2013, Giuliano et al., 2011, Hariri et al., 2013, Markowitz et al., 2013). The three available HPV vaccines each protect against two “high-risk” HPV types (HPV16 and 18) associated with the majority of HPV-related cancers. The quadrivalent and 9-valent vaccines also protect against two “low-risk” types (HPV6 and 11) associated with 90% of genital warts. The 9-valent vaccine protects against five additional “high-risk” types (HPV31, 33, 45, 52, and 58) responsible for 10% of HPV-related cancers (Petrosky et al., 2015). While the three-dose vaccine series may be initiated as early as age nine (Centers for Disease Control and Prevention, 2010), the Advisory Committee on Immunization Practices (ACIP) has recommended that the vaccine be routinely administered to 11–12-year-old females and males (quadrivalent and 9-valent only) since 2006 and 2011, respectively. However, only 60% of female and 41.7% of male 13–17-year-olds have received at least one dose of the vaccine as of 2014 (Reagan-Steiner et al., 2015). Limited research exists regarding rates of vaccine uptake at ages 9–12.

In addition to socioeconomic status, race/ethnicity, and general and vaccine-specific healthcare utilization behaviors (Centers for Disease Control and Prevention, 2013, Kessels et al., 2012, Reiter et al., 2013), healthcare provider recommendation appears to be a key factor in parents' decision to vaccinate their children against HPV (Dorell et al., 2013, Holman et al., 2013, Stokley et al., 2014, Donahue et al., 2014). However, most studies of predictors of vaccine uptake have focused on adolescents ages 13 and older. Greater understanding of factors influencing initiation prior to and during the recommended age range for routine HPV vaccination may identify potential targets for intervention to increase vaccination coverage.

Our objective was to explore rates and predictors of HPV vaccine initiation among 9–13-year-old males and females in the United States. Because limited data are available regarding HPV vaccine initiation prior to age 13, one aim of the study was to estimate the vaccine initiation rate among 9–12-year-olds. We were also able to compare our observed rate of initiation by age 13 to that of previously published national estimates for 13-year-olds from the same time period. An additional aim of the study was to examine characteristics of “early” initiators (i.e., individuals receiving at least one dose of the HPV vaccine by ages 9–10, prior to the targeted age for routine recommendation) as well as predictors of initiation among children who had already reached the targeted age range for routine administration (i.e., ages 11 and up).

## Methods

### Sample

Data were collected in August 2014 as part of a larger Web-based survey assessing attitudes and behaviors related to HPV and influenza vaccination in a national sample of mothers of 9–13-year-olds in the United States. The study was approved by the IRB at Indiana University. Data collection was facilitated by Survey Sampling International (SSI), a survey research company that maintains national panels of adults in 37 countries. Each panel member may participate in up to four surveys annually, and participants are entered into a lottery to win a monetary prize through SSI. E-mail invitations were sent at random to members of SSI's U.S. panel meeting the study's target demographic. Initially, 3208 panelists responded to a generic e-mail invitation to participate in a survey, with 2860 women (89%) agreeing to complete the survey after being presented with a brief description of the study. Of those agreeing to participate, 2446 (86%) met the eligibility criteria for participation (i.e., they were 18 years of age or older and the mother or female legal guardian of at least one 9–13-year-old child who lived in their household). Participants with more than one 9–13-year-old child were prompted to answer questions about their youngest child in this age range. Although the participants were recruited nationally, the sample does not constitute a nationally representative sample.

### Measures

We assessed HPV vaccination history using mother report of the number of doses of the HPV vaccine received by the child. Children who had received at least 1 dose of the vaccine were categorized as HPV vaccine initiators.

Additional items related to HPV vaccination included mother report of whether the child's healthcare provider had discussed with her that her child could receive the HPV vaccine, and, if so, the strength of recommendation (i.e., “In your opinion, how strongly did your child's healthcare provider recommend that that your child receive the HPV vaccine?”), which was reported on a 5-point scale ranging from strongly discouraged to strongly recommended. These two items were combined and collapsed into four categories: “did not discuss,” “no recommendation” (combining responses of “neither recommended nor discouraged,” “discouraged,” or “strongly discouraged” due to low frequency of individual categories), “recommended,” and “strongly recommended.” Mothers also reported whether the target child had any older siblings and whether any of these siblings had received the HPV vaccine.

Mothers responded to five items pertaining to general beliefs about the benefits of immunization (e.g., “It is important that people get vaccinated so that they can protect their health”) using a 5-point response scale (Rickert et al., 2014) (Chronbach's alpha = .78), with a higher score on each item reflecting stronger beliefs about the benefits of vaccination. The scale reflects the perceived benefits construct of the Health Beliefs Model (Skinner et al., 2015), with items chosen based on previous reports assessing factors influencing parents' immunization of early adolescents (Rickert et al., 2014). "Perceived benefits of vaccination" was included as a continuous variable in our analyses, calculated using the mean of the five items.

Mothers reported on multiple healthcare-utilization behaviors such as whether or not the child had visited a doctor, nurse, or other healthcare provider in the past year; whether the child has a regular healthcare provider (defined as a healthcare provider who knows the child and his/her health history), the type of location where the child typically visits a healthcare provider; and whether the child received the influenza vaccine during the most recent influenza season (approximately September 2013 to March 2014).

Sociodemographic characteristics included child's age, gender, race/ethnicity, geographic region, and health insurance type, as these variables have previously been associated with disparities in adolescent HPV vaccination.

### Statistical analyses

Children whose mothers reported uncertainty regarding the number of doses received or for whom vaccination history was missing (*n* = 261) were excluded from analyses predicting vaccine HPV initiation, resulting in a total sample size of 2185, or 89.3% of eligible participants. HPV vaccination data were more likely to be missing among target children (*n* = 261) who were male, belonged to racial/ethnic minorities, and did not have private insurance, and also more likely to be missing among 9-year-olds and less likely among 13-year-olds. No differences were found with regard to geographic region.

First, we compared HPV vaccine initiators to non-initiators with regard to categorical variables using chi-square tests of independence. We conducted a t-test of independent samples to assess whether there were differences between initiators and non-initiators with regard to mothers' perceived vaccine benefits. All variables found to have significant bivariate associations with initiation were then included in a multivariate logistic regression model comparing initiators to non-initiators. Lastly, we used chi-square tests of independence to examine differences between initiators and non-initiators separately among two age groups: 9–10-year-olds (those younger than the age range targeted for routine recommendation) and 11–13-year-olds.

## Results

Descriptive statistics for all variables are presented in Table 1. Frequencies and means correspond to the sample of 2185 participants providing initiation data. Of these participants, 34.9% had initiated the vaccine series (i.e., one or more doses had been received).

### Comparing HPV vaccine initiators and non-initiators

Table 1 presents the results of chi-square tests of independence comparing HPV vaccine initiators and non-initiators with *α* = .05. A significantly higher percentage of initiators were age 12 or 13, whereas a significantly higher percentage of non-initiators were age 9, 10, or 11. A significantly higher percentage of initiators were female, belonged to a racial/ethnic minority, had public health insurance or were uninsured, had an older sibling who received the HPV vaccine, received the flu vaccine during the most recent flu season, had visited a healthcare provider in the past year, and typically received healthcare services in a location other than a private office. Mothers of initiators reported significantly greater perceived general benefits of vaccination. A significantly higher percentage of initiators had mothers who reported that their child's healthcare provider had recommended or strongly recommended HPV vaccination, while a significantly higher percentage of non-initiators had mothers who reported that their child's healthcare provider had not discussed the possibility of vaccinating their child against HPV.

All variables found to have significant bivariate associations with initiation were then included in a logistic regression model predicting vaccine initiation. Table 2 presents odds ratios representing the effect of each measure in univariate models (Table 2, Model 1) followed by a multivariate model (Table 2, Model 2). In the multivariate model, relative to mothers who reported that a healthcare provider did not discuss the HPV vaccine with them, mothers were more likely to report initiation if they reported that their child's healthcare provider (a) discussed but did not specifically recommend vaccination, OR = 8.97 (5.74–14.00), (b) recommended vaccination, OR = 21.88 (15.38–31.12), or (c) strongly recommended vaccination, OR = 38.60 (26.61–56.00). Increased odds of initiation were also found with every one-year increase in age, OR = 1.23 (95% CI 1.13–1.35); having an older sibling who received the HPV vaccine, OR = 2.52 (1.94–3.27); and having received the flu vaccine last flu season, OR = 2.51 (1.91–3.31). However, decreased odds of initiation were found when receiving services in a private office, OR = .39 (.28–.54) for those with private insurance, OR = .72 (.55–.94), and for females, OR = .76 (.59–.98). Minority status, perceived benefits of vaccination, and past-year healthcare provider visit were not significant predictors of initiation in the multivariate model.

**Fig. 1:**
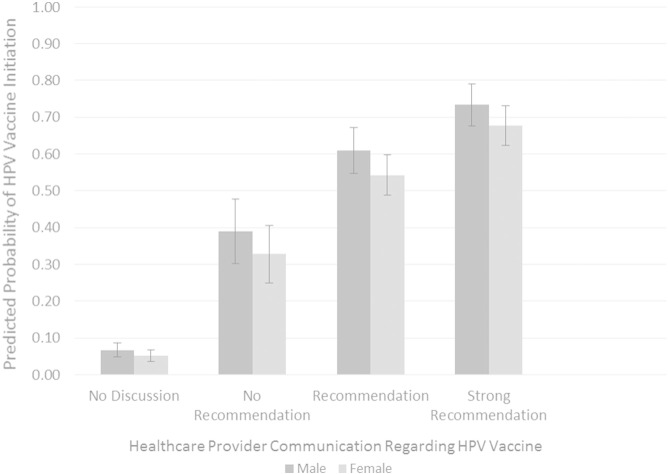
Predicted probability of HPV vaccine initiation by child gender and healthcare provider communication regarding HPV vaccination. Predicted probabilities were estimated based on multivariate logit models including variables shown in Table 2. Estimated values of all remaining variables held at their means. Error bars represent the 95% confidence interval of the predicted probability. Data were collected via a Web-based survey in 2014.

Because observed data suggest a substantial gender difference in vaccination, with females more likely to initiate vaccination than males, we conducted additional exploratory analyses to clarify the negative effect of female gender in our multivariate logistic regression model. We found that this effect was likely due to the association between gender and provider communication—when included in the multivariate logistic regression model, a gender × communication interaction term was statistically significant. To illustrate the interaction between child gender and healthcare provider communication (Fig. 1), we estimated the predicted probability of HPV vaccine initiation as a function of gender at each level of provider communication using estimates obtained from multivariate logit regression models and including all remaining variables from the multivariate model in Table 2 (Long and Freese, 2005). The pattern observed in Fig. 1 suggests that as the strength of provider recommendation increased, the predicted probability of initiation also increased. Provider discussion of HPV vaccination, regardless of recommendation, appeared to have a stronger effect on initiation among males than among females. The predicted probability of initiation without discussion for females (.05) was significantly lower than for males (.07, 95% CI for difference: .001, .030.) The predicted probability of initiation was significantly higher for males than for females following discussion without recommendation (.39 vs. .33, 95% CI for difference: .003, .122), recommendation (.61 vs. .54, 95% CI for difference: .005, .129), and strong recommendation (.73 vs. .68, 95% CI for difference: .004, .109).

Chi-square tests of independence also suggested significant differences in healthcare provider communication by gender (*χ*^2^ = 40.13, *df* = 3, *p* < .001). Among mothers of daughters, 41.2% (*n* = 503) reported that no discussion about HPV vaccination occurred; 8.6% (*n* = 105) reported that the HPV vaccine was discussed but either discouraged or not recommended; and 26.3% (*n* = 321) and 23.9% (*n* = 291) reported that their child's healthcare provider recommended or strongly recommended HPV vaccination, respectively. Among mothers of sons, 54.2% (*n* = 485) reported that no discussion about HPV vaccination occurred; 8.9% (*n* = 80) reported that the HPV vaccine was discussed but either discouraged or not recommended; and 20.4% (*n* = 183) and 16.4% (*n* = 147) reported that their child's healthcare provider recommended or strongly recommended HPV vaccination, respectively.

### Comparing HPV vaccine initiators and non-initiators by age group

Initiation was reported by 27.5% of 9–10-year-olds and 42.4% of 11–13-year-olds. We examined factors associated with HPV vaccine initiation vs. non-initiation by ages 9–10 as well as initiation vs. non-initiation by ages 11–13 (Table 3) using chi-square tests of independence, *α* = .05. A gender difference was found in initiation by ages 11–13 but not by ages 9–10. Because existing research shows that there are significant gender differences in HPV vaccination among older adolescents, we conducted additional chi-square tests to explore whether the rate of initiation differed by gender at each age within the target age range. No significant differences were found with regard to the rates of initiation between females and males by age 9 (25.4% vs. 26.0%, respectively, *p* = .88), age 10 (32.7% vs. 26.4%, *p* = .15), age 11 (29.8% vs. 31.4%, *p* = .74), or age 12 (54.1% vs. 44.4%, *p* = .06). By age 13, a significantly higher proportion of females had initiated vaccination (53.8%, versus 40.7% of males, *p* = .02).

Chi-square tests of independence also indicated differences in health insurance type by initiation status among 9–10-year-olds, but not among 11–13-year-olds, with a significantly greater percentage of those who had initiated the vaccine by ages 9–10 either receiving publicly funded health insurance or being uninsured (45.8%) compared to non-initiated 9–10-year-olds (39.8%). A significantly higher percentage of those initiated by ages 11–13 had visited a healthcare provider in the past year (96.5%) compared to those not initiated by ages 11–13 (91.4%), while this difference was not observed among 9–10-year-olds. Those initiating by ages 11–13 also had mothers who reported significantly higher perceived benefits of vaccination than those not initiating by ages 11–13, M(SD) 3.68 (.75) vs. 3.45 (.81).

## Discussion

We explored HPV vaccine initiation among 9–13-year-olds in the United States. To our knowledge, this is one of the first studies subsequent to the ACIP recommendation for routine immunization in early adolescence that assesses rates of HPV vaccine initiation among both males and females in this age range. This study adds to literature suggesting that provider communication and/or recommendation regarding HPV vaccination is a key factor in parents' vaccination decisions, suggesting that this may also be a key factor at earlier ages. We were also able to explore potential differences between children who initiated vaccination prior to and during the targeted ages for routine recommendation.

Our multivariate analyses indicated that child age, provider communication regarding HPV vaccination, flu vaccine history, sibling receipt of the HPV vaccine, health insurance type, and typical location of provider visits were significant predictors of initiation among 9–13-year-olds. Older age was associated with increased likelihood of having initiated the vaccine series, which is in keeping with previous estimates of coverage across later adolescence (Stokley et al., 2014, Dempsey et al., 2010, National Center for Health Statistics and National Health Interview Survey, 2010). The results of our study suggest that this effect is also present in the early adolescent period. Higher initiation rates across adolescence occur in conjunction with an increase in healthcare provider recommendation of vaccination across the age range (Vadaparampil et al., 2011, Roland et al., 2014, Weiss et al., 2010, Malo et al., 2014). Our results suggest that encouraging provider recommendation of vaccination at earlier ages could result in increased initiation rates at earlier ages, as discussed below. Additionally, parents who opt to vaccinate their children against the seasonal flu may be more accepting of other non-mandated immunizations such as the HPV vaccine, and those who have older vaccinated children may also be more accepting of the HPV vaccine for their younger children. High acceptance of HPV vaccination may be more common among parents of children receiving care outside of private clinics and/or who lack private health insurance benefits (Zimet et al., 2005). Again, our findings extend previous research among older adolescents into the younger end of the vaccine-eligible range. Interestingly, mothers' perceived benefits of vaccination, while associated with likelihood of initiation in bivariate analyses, were no longer a significant predictor of initiation in the multivariate analysis, suggesting that other factors may ultimately be more salient to parents' decisions regarding immunization.

Healthcare provider communication regarding HPV vaccination was a particularly strong predictor of initiation across our sample. This finding regarding younger children is consistent with previous research among parents of older adolescents indicating the significant impact of provider recommendation on parents' decision making surrounding HPV vaccination (Dorell et al., 2013, Holman et al., 2013, Stokley et al., 2014, Donahue et al., 2014, Alexander et al., 2014).. Unfortunately, almost half of mothers in our sample reported that their child's healthcare provider did not discuss HPV vaccination. When discussion did occur, however, it most commonly involved a recommendation or strong recommendation; Fig. 1 suggests that the majority of mothers would elect to vaccinate their son or daughter if it was recommended. Provider recommendation may increase parent perception of the vaccine as safe and effective, leading to increased rates of initiation (Staras et al., 2014).

Our finding that initiation among males may be more strongly affected by healthcare provider recommendation could reflect the relative recency of the ACIP's routine recommendation for males compared to females and the associated barriers to vaccination, such as less awareness of the importance of male vaccination among providers as well as among parents of sons. Such barriers are commonly reported by parents of sons, who may be willing to vaccinate once such barriers are addressed (Donahue et al., 2014).

Interestingly, the gender gap in initiation did not begin to emerge in our sample until around ages 12 to 13. Similarities in initiation rates among males and females at younger ages may reflect a trend toward increased availability and equality of administration of the vaccine for males, as licensure and routine recommendation of the vaccine for males (2009 and 2011, respectively) occurred more recently than for females (2006 and 2007, respectively).

Although the rates were similar among both genders at ages 11–12, coverage remains discouragingly low given that this is the age range in which the vaccine should be routinely administered. Missed opportunities for vaccination are frequent—the Centers for Disease Control and Prevention estimates that 91.3% of females born in 2000 would have received at least one dose of the HPV vaccine by age 13 if it had been administered during healthcare visits when they received another immunization (Stokley et al., 2014). In our own sample, 92.3% of 9–13-year-olds who had not yet initiated the HPV vaccine had seen a healthcare provider within the past year, highlighting the importance of healthcare provider communication regarding vaccination whenever such an opportunity arises. In conjunction with previous research, our findings suggest that provider-focused interventions aimed at promoting communication about the HPV vaccine may reduce such missed opportunities and increase HPV vaccination coverage among youth (Perkins et al., 2015). Improving provider communication about the HPV vaccine during the early adolescent age range may be particularly beneficial, as older adolescents tend to seek preventive care services less frequently (Rand et al., 2007) and are also at greater risk for experiencing HPV exposure prior to vaccination.

The study has several limitations. First, data were not collected from a nationally representative sample. However, the demographics of our sample are comparable to the most recent available U.S. census estimates pertaining to the distribution of individuals across geographic regions (U.S. Census Bureau) as well as the distribution of racial groups among 9–13-year-olds (U.S. Census Bureau, 2015a, U.S. Census Bureau, 2015b). Second, parental recall of vaccination history rather than immunization records may produce accurate estimates of overall coverage but less accurate estimates among racial minorities or individuals of lower socioeconomic status (U.S. Census Bureau, 2015a, U.S. Census Bureau, 2015b). However, mother-reported vaccination status may be more accurate than reports from other caregivers (Ojha et al., 2013, Attanasio and McAlpine, 2014). Of note, the initiation rate among 13-year-olds in our 2014 sample (females, 53.8%; males, 40.7%) is similar to coverage estimates for 13-year-olds in the 2014 NIS-Teen sample (females, 51.1% ± 4.1; males, 38.9% ± 4.2), confirmed via vaccination records (Reagan-Steiner et al., 2015). Third, it is possible that parents who initiated vaccination and/or have more favorable attitudes toward vaccination may be more likely to recall having received positive provider communication about the vaccine. Similarly, healthcare providers familiar with a family's vaccination attitudes and history may be more likely to recommend the vaccine to vaccine-friendly parents, and parents may seek out providers with whom they share opinions about the importance of immunization practices. Fourth, we did not assess age at initiation, meaning that some individuals categorized as having initiated the vaccine by ages 11–13 may have in fact initiated by ages 9–10, which would result in an underestimation of individuals as early initiators. Finally, data were unavailable regarding panel members who viewed the initial e-mail invitation for participation and opted not to participate, which prevented us from identifying possible patterns of characteristics among nonresponders.

## Conclusions

In one of the first studies to assess rates of HPV vaccine initiation among 9–13-year-olds following the ACIP recommendation for routine immunization in early adolescence, we found that one quarter of 9–10-year-olds had initiated the vaccine series, with coverage increasing across the age range, and no gender differences in initiation at younger ages. Strength of health provider recommendation to parents about HPV vaccination emerged as a key factor in parents' decision to vaccinate their children. Given the ubiquity of HPV and the vaccine's effectiveness against adverse health consequence, the frequency of initiation occurring prior to the targeted age for routine administration holds promise; however, vaccination coverage among early adolescents remains well below national health goals. Improvements in provider communication with patients and their parents could substantially contribute to HPV vaccination during early adolescence becoming truly routine.

## Conflict of interest statement

Gregory Zimet has been an investigator on investigator-initiated research funded by Merck, Inc., has served as a consultant to Merck, Inc., and has received an unrestricted program development grant from GlaxoSmithKline. The other authors have no conflicts of interest to disclose. The funding organizations did not play a direct role in the design and conduct of the study; management, analysis, or interpretation of the data; preparation, review, or approval of the manuscript; or the decision to submit the manuscript for publication.

## Figures and Tables

**Table 1:** Sample characteristics of 9–13-year-olds in the U.S. who have and have not initiated HPV vaccination.

	Full sample	Initiated^a^	Not initiated	
Measure	(*n* = 2185)	(*n* = 763)	(*n* = 1422)	*p* value
Age, *n* (%)				
9	635 (29.1)	163 (21.4)^f^	472 (33.2)^f^	< .001
10	454 (20.8)	136 (17.8)^f^	318 (22.4)^f^	
11	398 (18.2)	121 (15.9)^f^	277 (19.5)^f^	
12	389 (17.8)	194 (25.4)^f^	195 (13.7)^f^	
13	307 (14.1)	149 (19.5)^f^	158 (11.1)^f^	
Gender, *n* (%)				
Female	1257 (57.5)	464 (60.8)	793 (55.8)	.02
Male	928 (42.5)	299 (39.2)	629 (44.2)	
Race/ethnicity, *n* (%)^b^				
Minority or multiracial/ethnic	720 (33.8)	303 (40.7)	417 (30.0)	< .001
White	1413 (66.2)	441 (59.3)	972 (70.0)	
Geographic region, *n* (%)^c^				
Northeast	391 (18.3)	141 (18.9)	250 (17.9)	.35
Midwest	500 (23.4)	176 (23.6)	324 (23.3)	
South	814 (38.1)	265 (35.5)	549 (39.4)	
West	433 (20.2)	164 (22.0)	269 (19.3)	
Puerto Rico	1 (.0)	0 (.0)	1 (.1)	
Health insurance type, *n* (%)				
Private	1298 (59.4)	426 (55.8)	872 (61.3)	.01
Public/self-pay	887 (40.6)	337 (44.2)	550 (38.7)	
Provider communication about HPV vaccine, *n* (%)				
Did not discuss	989 (46.7)	69 (9.0)^f^	929 (69.5)^f^	< .001
No recommendation^d^	185 (8.7)	65 (8.5)	120 (8.4)	
Recommended	504 (23.8)	302 (39.6)^f^	202 (14.2)^f^	
Strongly recommended	438 (20.7)	327 (42.9)^f^	111 (7.8)^f^	
Sibling HPV vaccination status, *n* (%)				
Received	627 (29.2)	356 (47.5)	271 (19.4)	< .001
Did not receive/no older sibling	1519 (70.8)	393 (52.5)	1125 (80.6)	
Flu vaccine in most recent flu season, *n* (%)				
Received	1284 (59.1)	573 (75.4)	711 (50.4)	< .001
Not received	887 (40.9)	187 (24.6)	700 (49.6)	
Perceived benefits of vaccination, mean (SD)	3.54 (.79)	3.61 (.74)	3.50 (.81)	.002
Doctor, nurse, or other healthcare provider visit in past year, *n* (%)				
Yes	2042 (93.6)	731 (96.1)	1311 (92.3)	.001
No	139 (6.4)	30 (3.9)	109 (7.7)	
Regular healthcare provider, *n* (%)				
Yes	2087 (95.8)	737 (96.7)	1350 (95.3)	.11
No	92 (4.2)	25 (3.3)	67 (4.7)	
Service location, *n* (%)				
Private office	1744 (79.8)	540 (70.8)	1204 (84.7)	< .001
Other location^e^	441 (20.2)	223 (29.2)	218 (15.3)	

Note: Data were collected via a Web-based survey in 2014.

a Among vaccine initiators, dose completion reported as follows: 1 dose, *n* = 283; 2 doses, *n* = 212; 3 doses, *n* = 268.

b Child race/ethnicity was self-designated by participants as follows: African American/Black, 14.3%; American Indian, 2.3%; Asian, 4.7%; Hispanic or Latino, 13.0%; White, 75.2%; Native Hawaiian or other Pacific Islander, .9%; Other, 1.8%; No response, .3%. Mothers were asked to select multiple race/ethnicities when applicable. Participants were categorized as belonging to a racial/ethnic nonminority (i.e., White) or minority, (including 21.0% of participants who reported a single minority race/ethnicity and 12.8% who indicated multiple race/ethnicities).

c Geographic location was determined from mother's reported zip code and categorized based on the U.S. census region.

d Category includes mothers reporting that the vaccine was “neither recommended nor discouraged,” *n* = 174, “discouraged,” *n* = 9; or “strongly discouraged,” *n* = 2.

e Includes community health clinic, university-based health clinic, emergency room or urgent care clinic, or other

f For variables consisting of more than 2 categories, denotes significant group differences for that category at *p* < .05.

**Table 2:** Odds ratios (OR) from univariate (Model 1) and multivariate (Model 2) logistic regression models predicting HPV vaccine initiation among 9–13-year-olds.

	Model 1			Model 2		
Variable	OR	95% CI	*p* value	OR	95% CI	*p* value
Age	1.33	(1.25–1.40)	< .001	1.23	(1.13–1.35)	< .001
Gender						
Male (ref. category)	1.00			1.00		
Female	1.23	(1.03–1.47)	.02	.76	(.59–.98)	.04
Race/ethnicity						
Minority or multiracial/ethnic (ref. category)	1.00			1.00		
White	.62	(.52–.75)	< .001	.84	(.65–1.09)	.19
Health insurance type						
Public/self-pay (ref. category)	1.00			1.00		
Private health insurance	.80	(.67–.95)	.01	.72	(.55–.94)	.01
Provider communication regarding HPV vaccine						
Did not discuss (ref. category)	1.00			1.00		
No recommendation given	8.39	(5.63–12.50)	< .001	8.97	(5.74–14.00)	< .001
Recommendation given	23.15	(16.88–31.75)	< .001	21.88	(15.38–31.12)	< .001
Strong recommendation given	45.61	(32.52–63.98)	< .001	38.60	(26.61–56.00)	< .001
Sibling HPV vaccination status						
Did not receive/no older sibling (ref. category)	1.00			1.00		
Older sibling received HPV vaccine	3.76	(3.09–4.56)	< .001	2.52	(1.94–3.27)	< .001
Flu vaccine in most recent flu season						
Did not receive (ref. category)	1.00			1.00		
Received	3.02	(2.48–3.67)	< .001	2.51	(1.91–3.31)	< .001
Perceived benefits of vaccination	1.19	(1.07–1.34)	.002	1.04	(.88–1.23)	.64
Doctor, nurse, or other healthcare provider visit in past year						
No (ref. category)	1.00			1.00		
Yes	2.03	(1.34–3.07)	.001	.86	(.47–1.56)	.61
Location where typically receives services						
Other location (ref. category)	1.00			1.00		
Private clinic	.44	(.35–.54)	< .001	.39	(.28–.54)	< .001

Note: Data were collected via a Web-based survey in 2014.

**Table 3:** Comparison of HPV vaccine initiation status by ages 9–10 and 11–13.

	Vaccination status by ages 9–10			Vaccination status by ages 11–13		
	Initiated	Not Initiated		Initiated	Not Initiated	
Measure	(*n* = 299)	(*n* = 790)	*p* value	(*n* = 464)	(*n* = 630)	*p* value
Gender, *n* (%)						
Female	173 (57.9)	434 (54.9)	.39	291 (62.7)	358 (56.8)	.05
Male	126 (42.1)	356 (45.1)		173 (37.3)	272 (43.2)	
Race/Ethnicity, *n* (%)						
Minority or multiracial/ethnic	121 (41.4)	230 (29.9)	< .001	182 (40.3)	186 (30.1)	.001
White	171 (58.6)	540 (70.1)		270 (59.7)	431 (69.9)	
Geographic region, *n* (%)						
Northeast	52 (17.8)	136 (17.6)	.23	89 (19.6)	114 (18.4)	.90
Midwest	70 (20.4)	170 (22.0)		106 (23.3)	154 (24.9)	
South	103 (35.3)	325 (42.0)		162 (35.7)	224 (36.2)	
West	67 (22.9)	141 (18.2)		97 (21.4)	126 (20.4)	
Puerto Rico	0 (.0)	1 (.1)		0 (.0)	0 (.0)	
Health insurance type, *n* (%)						
Private	162 (54.2)	483 (61.1)	.04	264 (56.9)	388 (61.6)	.12
Public/self-pay	137 (45.8)	307 (38.9)		200 (43.1)	242 (38.4)	
Provider communication about HPV vaccine, *n* (%)						
Did not discuss	32 (11.0)^a^	589 (76.8)^a^	< .001	28 (6.1)^a^	339 (57.2)^a^	< .001
Discouraged/No recommendation	18 (6.2)	43 (5.6)		47 (10.2)	77 (13.0)	
Recommended	113 (38.7)^a^	80 (10.4)^a^		189 (40.9)^a^	122 (20.6)^a^	
Strongly recommended	129 (44.2)^a^	55 (7.2)^a^		198 (42.9)^a^	55 (9.3)^a^	
Sibling HPV vaccination status, *n* (%)						
Received	144 (49.3)	142 (18.3)	< .001	212 (46.3)	128 (20.6)	< .001
Did not receive/no older sibling	148 (50.7)	632 (81.7)		246 (53.7)	492 (79.4)	
Flu vaccine in most recent flu season, *n* (%)						
Received	234 (78.5)	435 (55.5)	< .001	339 (73.4)	276 (44.2)	< .001
Not received	64 (21.5)	349 (44.5)		123 (26.6)	349 (55.8)	
Perceived benefits of vaccination, mean (SD)	3.50 (.71)	3.54 (.81)	.41	3.68 (.75)	3.45 (.81)	< .001
Doctor, nurse, or other healthcare provider visit in past year, *n* (%)						
Yes	284 (95.3)	735 (93.0)	.17	447 (96.5)	574 (91.4)	.001
No	14 (4.7)	55 (7.0)		16 (3.5)	54 (8.6)	
Regular healthcare provider, *n* (%)						
Yes	293 (98.0)	757 (96.1)	.12	444 (95.9)	591 (94.3)	.14
No	6 (2.0)	31 (3.9)		19 (4.1)	36 (5.7)	
Service location, *n* (%)						
Private office	205 (68.6)	673 (85.2)	< .001	335 (72.2)	530 (84.1)	< .001
Other location	94 (31.4)	117 (14.8)		129 (27.8)	100 (15.9)	

*p* value is for two-sided test, *α* < .05. Chi-square and t-tests were used to compare vaccine initiators and non-initiators with regard to categorical and continuous variables, respectively.

Note: Data were collected via a Web-based survey in 2014.

a For variables with more than two categories, this denotes significant group differences for specific category at *p* < .05.
